# Gut Microbiota, Host Organism, and Diet Trialogue in Diabetes and Obesity

**DOI:** 10.3389/fnut.2019.00021

**Published:** 2019-03-13

**Authors:** Veronica Lazar, Lia-Mara Ditu, Gratiela G. Pircalabioru, Ariana Picu, Laura Petcu, Natalia Cucu, Mariana Carmen Chifiriuc

**Affiliations:** ^1^Department of Microbiology and Immunology, Faculty of Biology, University of Bucharest, Bucharest, Romania; ^2^Earth, Environmental and Life Sciences Section, Research Institute of the University of Bucharest, University of Bucharest, Bucharest, Romania; ^3^National Institute for Diabetes, Nutrition and Metabolic Diseases Prof. Dr. N. Paulescu, Bucharest, Romania; ^4^Fundeni Clinical Institute, Bucharest, Romania; ^5^Department of Genetics, Faculty of Biology, University of Bucharest, Bucharest, Romania

**Keywords:** microbiome, gut physiology, diet, diabetes, obesity, prebiotics, probiotics

## Abstract

The gastrointestinal tract with its microbiota is a complex, open, and integrated ecosystem with a high environmental exposure. It is widely accepted that the healthy gut microbiotais essential for host homeostasis and immunostasis, harboring an enormous number and variety of microorganisms and genes tailored by hundreds of exogenous and intrinsic host factors. The occurrence of dysbiosis may contribute to host vulnerability and progression to a large spectrum of infectious and non-communicable diseases, including diabetes and obesity, two metabolic disorders that are showing an endemic trend nowadays. There is an urgent need to develop efficient strategies to prevent and treat metabolic disorders such as diabetes and obesity which are often associated with serious complications. In this paper, we give an overview on the implications of gut microbiota in diabesity, with a focus on the triangle gut microbiota—diet-host metabolism and on the way to manipulate the gut microbial ecosystem toward achieving novel diagnosis and predictive biomarkers with the final goal of reestablishing the healthy metabolic condition. The current research data regarding the precision/personalized nutrition suggest that dietary interventions, including administration of pre-, pro-, and syn-biotics, as well as antibiotic treatment should be individually tailored to prevent chronic diseases based on the genetic background, food and beverage consumption, nutrient intake, microbiome, metabolome, and other omic profiles.

## Introduction

**Figure 1:**
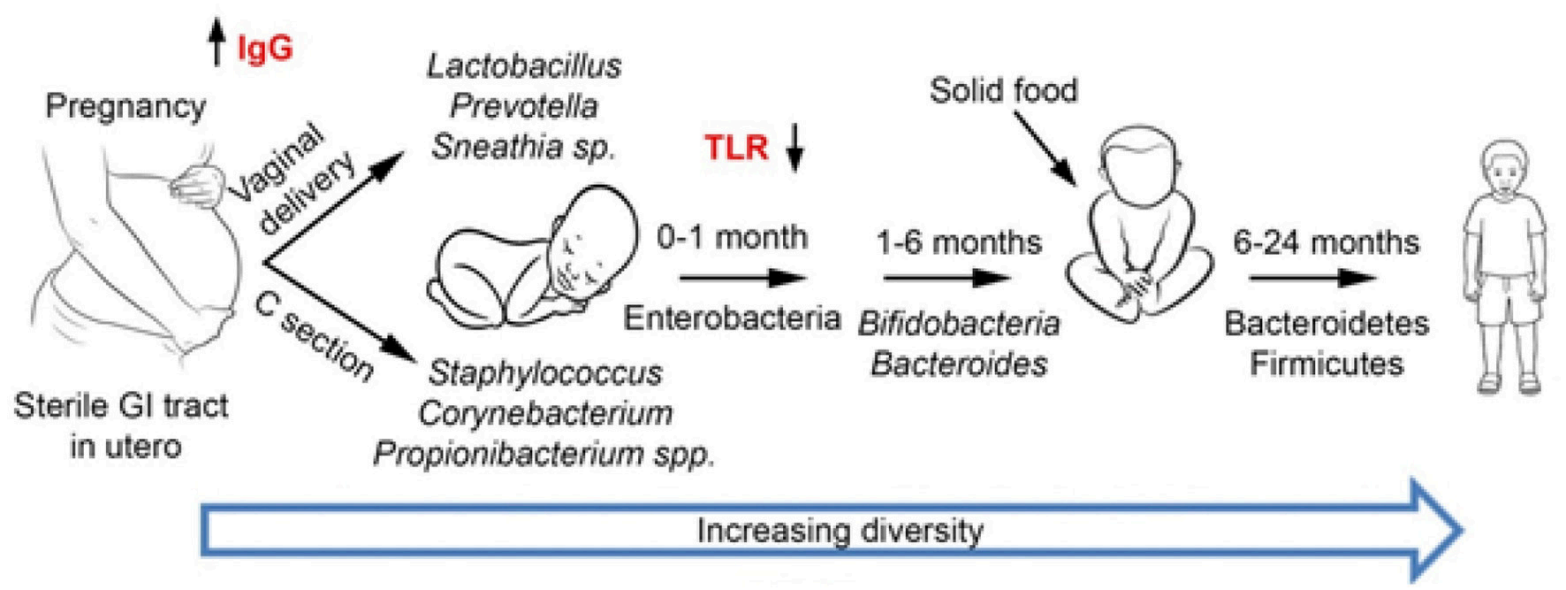
Development of gut microbiota. During the first years of life, the microbiota is largely influenced by external factors, such as delivery mode and type of feeding (breast or artificial formula feeding). Subsequently, the intake of solid food as well as the gradual maturation of the immune system modulates the gut microbiota. By the age of 2–3 years old, the microbiota resembles that of an adult with Bacteroidetes and Firmicutes as the main phyla.

Human microbiota includes bacteria, fungi, archaea, protozoans, and viruses, which seem to be even more numerous compared to those contained in the human genome (1). The microorganisms inhabiting the organism achieve a perfect mutual synergy with its host, being often referred together as a “superorganism” or a host extra-organ. The gastrointestinal tract (GIT) contains at least 10^14^ bacteria, with the highest density achieved in the large intestine, while the number of genes (intestinal microbiome) is superior (150- to 500-fold) to human DNA (2). Thus, GIT microbiota could be practically considered the fourth organ of the digestive system or the “forgotten organ” living in the gut like in a bioreactor (3). Large-scale projects, such as the European Metagenomics of the Human Intestinal Tract (MetaHIT) (4) and the US Human Microbiome Project Consortium(5) report more than 2,000 species, classified into 12 different phyla, of which 93.5% belong to Proteobacteria, Firmicutes, Actinobacteria, and Bacteroidetes (6–8). Independently of birth place, sex, age, or body weight, there are three predominant “enterotypes,” enriched in *Bacteroides, Ruminococcus*, and *Prevotella* (9). The GIT microbiota composition (diversity or the abundance of particular species) is shaped by hundreds of factors, including host genetics, mode of delivery (Figure 1), gender, age, height, weight, diet, immune system, gastrointestinal secretions blood levels of various molecules or red blood cell counts, stool consistency, sleep, medical history, ethno-geographical and socio-economic conditions, sanitary conditions, smoking, antibiotics and antibiotics-like substances, laxatives and less intuitive drugs (e.g., antihistamines, antidepressants, and metformin) (10–13). A deep sequencing study of the gut microbiomes revealed correlations between the microbiome and 126 exogenous and intrinsic host factors, including 12 diseases, 31 intrinsic factors, 19 drug groups, 60 dietary factors, and 4 smoking categories (10).

## Role of GIT Microbiota in the Host Energy Balance

**Figure 2:**
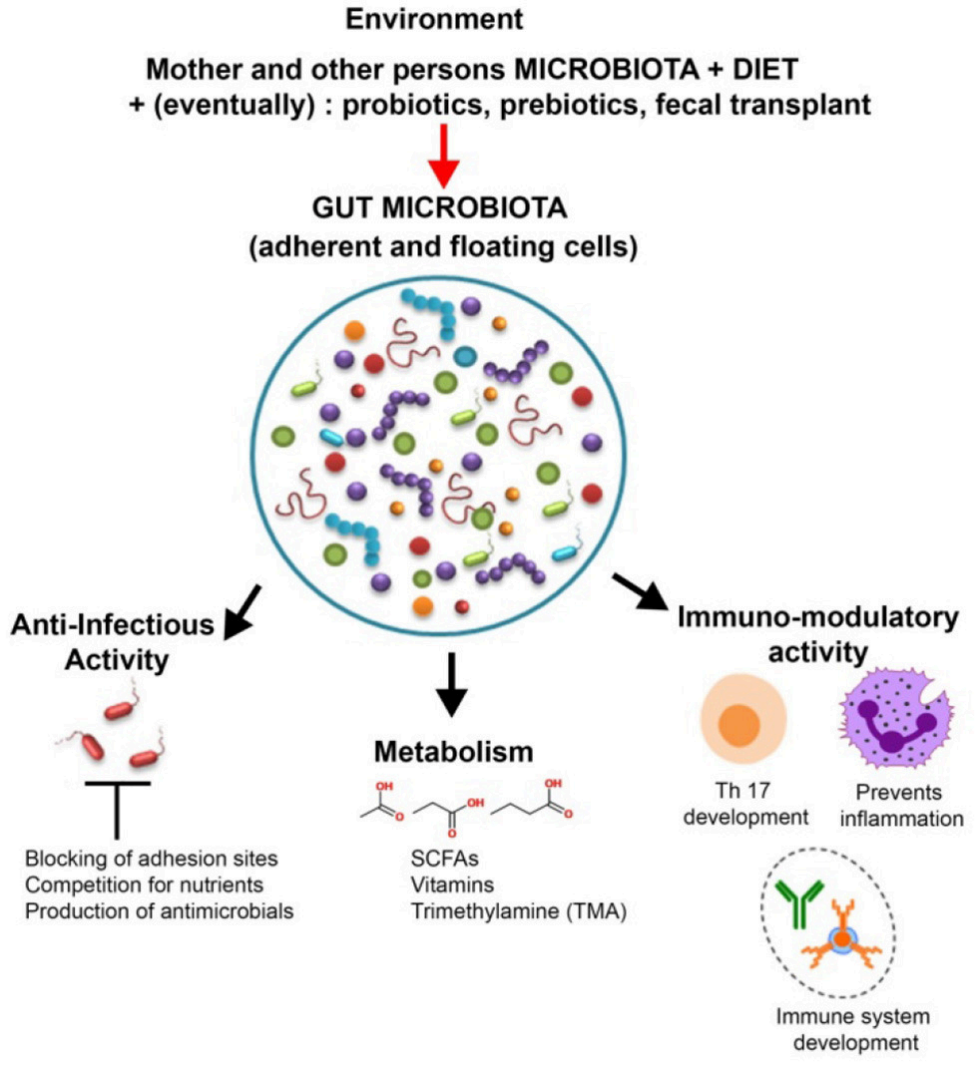
Roles and modulation of gut microbiota. In addition to helping digestion and synthesizing vitamins and other metabolites, such as short-chain fatty acids (SCFAs), the members of the gut microbiota play an important role in host defense (by producing antimicrobial compounds and competing against pathogens for adhesion sites and nutrients) as well as in the development and training of the immune system. The gut microbiota is influenced by a wide array of factors such as diet, probiotics, and antibiotics.

GIT microbiota plays a significant role in human health and disease (1) (Figure 2). The microbiota is a major player in energy harvest and storage, as well as in a variety of metabolic functions, such as bile acids and choline transformation, fermenting and absorbing undigested carbohydrates or providing vitamins and amino acids for the host (14).

Recent studies show that the microbiota may impact weight-gain and adiposity *via* several inter-connected pathways, such as energy harvest and production of microbial metabolites, through effects on inflammatory responses and on the gut-brain axis.

One of the most important metabolic activity of GIT microbiota is the production of non-gaseous SCFAs (acetate, propionate, and butyrate), through fermentation of microbiota-accessible, complex carbohydrates (MAC) (e.g., oligosaccharides, resistant starch, and plant cell wall materials) (15–17). The predominant commensal bacteria that produce SCFAs are represented by *Akkermansia muciniphilia, Prevotella* spp., *Ruminococus* spp., *Coprococcus* sp., *Faecalibacterium prausnitzii, Eubacterium rectale*, and *Roseburia* spp. (18). Absorbable SCFAs are important modulators of gut health and immune function (19), intestinal hormone production, and lipogenesis (20). SCFAs can interact with the host through many pathways. SCFAs signal through G-protein-coupled receptors such as G-protein coupled receptor GPR41 and GPR43 which affect crucial processes (e.g., inflammation, expression of tight junction proteins, and enteroendocrine regulation) and have a crucial role in maintaining an acid pH favoring the proliferation of certain bacterial species (16, 21, 22). Propionate, butyrate, and acetate trigger the local release of peptide YY (PYY) and of glucagon-like neuropeptide-1 (GLP-1) from enteroendocrine L cells regulating digestion and alter the liver function by modulating lipid metabolism with an indirect effect on the storage of fatty acids in the liver. Butyrate in particular is an energy substrate for colonocytes, releasing 1,000 kcal/day. Due to the trophic role on the intestinal epithelium and by promoting GLP-2 release and increasing mucus secretion, butyrate decreases the permeability of the intestinal barrier and is protective against colitis and colorectal cancers. SCFAs pathways were shown to be elevated in obesity metagenomic studies, and SCFAs levels were higher in overweight or obese people and animal models. Propionate is a substrate for gluconeogenesis, which signals through the central nervous system and protects the host from diet-induced obesity and glucose intolerance. Increased levels of propionate were associated with the microbiota following gastric bypass, which granted protection from diet-induced obesity upon transfer to germ-free recipient mice (2, 18).

Gut microbial metabolism of choline (a water-soluble nutrient essential for human life, found in eggs, seafood, beef, turkey and chicken, tuna, salmon, code and sardines, broccoli, cauliflower, cabbage, spinach, green peas, mushrooms, tomatoes etc.) leads to accumulation of trimethylamine (TMA), which is converted in the host liver to trimethylamine-*N*-oxide (TMAO) by flavin-monooxigenenase 3 (FMO3) (2). Circulating levels of TMAO were linked to an increased risk of cardiovascular diseases mortality and type 2 diabetes (T2DM) (23). Regulation of TMA-producing microbial species or their activity in the intestinal microbiota could aid to find novel means for preventing or treating atherosclerosis and choline deficiency-associated diseases. Resveratrol reduces TMAO levels through down-regulation of the enterohepatic farnesoid X receptor-fibroblast growth factor (FXR) axis, indicating that the gut microbiota is a potent target for personalized medicine interventions in diminishing metabolic disease development risk (24, 25). Another TMAO precursor, betaine is also widely distributed in animals (seafood), plants (spinach, wheat germ, bran) and microorganisms, mainly acting as an osmolyte (protecting cells and proteins from environmental stress) and methyl donor (transmethylation). Insufficient dietary levels of methyl groups results in hypomethylation in several metabolic pathways such as the hepatic fat metabolism, leading to steatosis and plasma dyslipidemia. Betaine was reported to protect internal organs, improving vascular risk factors, and enhancing performance, these beneficial effects justifying the efforts for the development of databases concerning betaine content in food (26).

Secondary bile acids are generated from cholesterol by microbiota of the lower small intestine and colon. By binding to distinct receptors, such as G-protein-coupled bile acid receptor 1 (TGR5) and the bile acid receptor FXR, secondary bile acids serve as signaling molecules. The ability to metabolize the naturally occurring FXR antagonist tauro-β-muricholic acid is an essential process toward obesity, steatosis, as well as impaired tolerance to glucose and insulin. Bariatric surgery is associated with changes in the metabolism of bile acids and in the microbiota composition. Germ-free mice that received a fecal transplant from people that had Roux-en-Y gastric bypass 10 years before, gained less fat compared to mice that were colonized by microbiota from obese people (2).

Importantly, the microbiota regulates tissue-level immune maturation *via* the microbial metabolism of tryptophan. Specifically, commensal *Lactobacilli* use tryptophan as an energy source to generate ligands of the aryl hydrocarbon receptor (AhR), a transcription factor involved in the organogenesis of intestinal lymphoid follicles (ILFs). AhR impacts IL-22 production and thus affect the secretion of anti-microbial peptides (lipocalin-2, S100A8, and S100A9) (27).

Other microbial metabolites whose functions in the host physiology and pathophysiology are still not yet elucidated include: indole propionic acid, associated with an improved epithelial barrier in the gut; ethylphenyl sulfate, correlated with the exacerbation of autistic behavior in a mouse model; indoxylsulfate and *p*-cresyl sulfate, both linked with poor cardiovascular outcomes in patients with uremia (2).

The brain signals to the gut through efferent vagal signaling and neuroendocrine pathways. The communication pathway with the microbiota is direct when neurotransmitters (5-hydroxytryptamine [5-HT], γ-aminobutyric acid [GABA], catecholamines) are sensed by the microorganisms, or indirect, *via* effects on the intestinal niche. The intestinal microenvironment can be modified by vagal efferent nerves involved in intestinal physiology (i.e., mucus secretion), mucosal immune responses, gut motility, intestinal barrier function, all of which impact microbiome composition and function (28).

The intestine signals to the brain *via* blood-borne substances or through afferent vagal and spinal nerves. Metabolites released by the microbiota can act as signaling molecules that regulate hormone {peptide YY [PYY], glucagon-like peptide-1 [GLP-1]} secretion from intestinal enteroendocrine cells. GLP-1 and PYY have receptors expressed in brain regions involved in the regulation of host energy balance (29).

### Host (Epi)genetics—Git Microbiota—Diet Metabolic Interplay

The gut microbiota of traditional rural populations in various parts of the world that have been separated for thousands of years on different continents showed increased bacterial diversity and presence of microbial taxa, lacking from the Western populations, suggesting that modern lifestyles, medical practices and processed foods in the industrialized world lead to an overall decline in gut microbiome biodiversity and loss of specific phylogenetic groups.

The microbiota contributes to the bioavailability, transformation, absorption, and/or excretion of several chemical elements including selenium, zinc, cobalt, and iodine all of which are co-factors for various enzymes involved in epigenetic processes (30).

Several studies proved an interplay between host genetics and diet in regulating the microbiome composition. Modifications in adiposity and metabolic response of low-calorie, weight-loss diets could be significantly altered by different genetic variants, especially those linked to T2DM, obesity, food preference and metabolism (23). A genome-wide significant variant in *LCT* region (*lactase gene*) modulating *Bifidobacterium* sp. abundance in the intestinal microbiome, associated with dairy intake was recently identified. A study in the elderly Mediterranean population revealed an association of the *LCT* variant and obesity, significantly regulated by dairy lactose and milk intake (23). Therefore, diet could alter the composition and/or abundance of the intestinal microbiota, as well as the microbial metabolome (31, 32). For instance, studies in Japanese populations have reported that after consumption of seaweeds, the genes encoding enzymes involved in metabolizing marine red algae were transferred from the marine-associated bacteria to certain bacteria in the intestinal microbiome (20). In fact, the microbiome has a huge role in extending our own metabolism for constituting the *hologenome*, defined as the sum of the host and its microbiota genetic information, representing the result of their longtime coevolution, finally achieving the host metabolic diversity (33, 34). Many bacterial enzymes, such as beta-glucosidase, beta-glucuronidase, nitroreductase, azoreductase, 7-alpha-dehydroxilase, cholesterol-dehydrogenase are inducible enzymes whose colon concentration is strongly influenced by the diet. Interestingly, omnivorous subjects had enhanced TMAO levels compared to vegans or vegetarians after ingesting L-carnitine, via an intestinal microbiota-dependent mechanism (20). There are reports of a gene-diet interaction in regulating *Bifidobacterium* sp. and other genera abundance, hence demonstrating the importance of host-microbiome interplay (17, 31).

Microbiota can promote epigenetic modification: the host responds to environmental factors through the alterations of DNA methylation and histone modifications. DNA methylation affects gene expression by regulating accessibility of the transcriptional machinery, transcription factors, and histone modifiers and to chromatin. DNA methyltransferases (DNMTs) can add a methyl group from the donor S-adenosylmethionine (SAM) to the carbon-5 position of the cytosine (5 mC), whereas the ten-eleven translocation enzyme (TET) dioxygenase family oxidizes 5 mC to hydroxymethylcytosine (5 hmC). Bacteria such as *Bifidobacterium* and *Lactobacillus* produce folate which supports the generation of SAM (35). Dietary methionine modulates the composition of the host microbiota as well as bacterial metabolism to release substrates for SAM synthesis. The mechanisms involved in microbiota-dependent modification of histones are still poorly understood. Acetylation of histones exposes target sites in nucleosomal DNA for transcription factors whereas deacetylation triggered by histone deacetylase (HDAC) removes acetyl groups from histone tails, hence leading to a reduction in transcriptional accessibility. Butyrate, produced by various commensal bacteria (*Faecalibacterium, Coprococcus, Roseburia, Eubacterium*, etc.) from dietary fiber has emerged as a HDAC inhibitor, with anti-inflammatory activity by suppressing STAT1 and NF-kB activation (36).

Several infectious agents (human papilloma virus, hepatitis viruses B and C, *Epstein–Barr* virus, polyomaviruses, *Chlamydia pneumoniae, Campylobacter rectus, Streptococcus bovis, Helicobacter pylori*) and members of the gut microbiota are epigenetic factors implicated in the metabolic syndrome pathogenesis. An example of an indirect action of microbial low molecular weight (LMW) molecules on chromatin remodeling, is the deficiency of some substrates (betaine, methionine, choline) and/or cofactors (vitamins B12, B2, B6, folate) generated by the microbiota. Indigenous intestinal bacteria may affect the bioavailability of dietary methyl groups and cause the hypomethylation of several epigenomic-associated pathways. This alteration may hinder DNA methylation leading to decreased SAM content, elevated plasma homocysteine concentrations, and elevated risk of various hepatica and vascular diseases, as well as malignancy. LMW molecules including SCFAs, sulforaphane cysteine/sulforaphane N-acetyl-cysteine and allylmercaptan/diallyldisulfide produced during the microbial metabolism of cruciferous vegetables or garlic could interfere with the activity of other enzymes responsible for epigenetic modifications, such as deacetylases, acetyltransferases, phosphotranferases, nucleases, serine-threonine protein kinases, etc. Also, gut microbiota is the main donor of acetyl groups for the formation of acetyl-CoA that is involved in epigenomic acetylation reactions. Bacteria and eukaryotes biosynthesise coenzyme A (CoA) from pantothenate, cysteine and β-alanine, all of which are found in most foods in small amounts and also produced by the gut microbiota. Deficiencies in these nutrients impair the synthesis of NADH, acetyl-CoA, and NAD, leading to disorders of the epigenomic acetylation machine involved in chromatin remodeling and post-translational protein modifications (30).

### Interplay of Factors Involved in Diabetes, Obesity, Oxidative Stress, And Inflammation

According to WHO report (37), the number of people with diabetes was four times higher in 2014 than in 1980, the majority living in developed countries and the WHO's prediction for 2035 is that diabetes will become the seventh cause of death worldwide, its prevalence increasing in low-income countries (37). The International Diabetes Federation (IDF) currently estimates that so far 415 million people worldwide have been diagnosed with T2DM and anticipate an increase up to 640 million by the year 2040 (38, 39). Diabesity describes the association between T2DM and obesity, both being a worldwide health problem, due to their extremely high prevalence and serious complications, particularly cardiovascular, respectively micro and macroangiopathy associated with accelerated vascular aging leading to atherosclerosis and microvascular dysfunction. According to the statistical data published by World Health Organization (WHO), in 2030 about a fifth of the world's adult population will suffer from metabolic syndrome and almost 23.6 million people will die from cardiovascular disorders (32). The interactions between genetic and environmental factors such as over-nutrition and a sedentary lifestyle lead to the development of these polygenic diet-related diseases with epidemic proportions (40, 41). Insulin resistance is also associated with an increased flux of free fatty acids (FFA) contributing to diabetic dyslipidemia, one of the major risk factors for cardiovascular disease in diabetes patients. Thus, an impaired glucose and lipid metabolism is the hallmark of metabolic syndrome, defined by central (abdominal) obesity and the presence of two to four of the following factors—reduced high density lipoprotein (HDL) cholesterol, high blood pressure, elevated triglycerides, higher resting metabolic rates (42) and increased fasting blood glucose. Obesity adds to the genetic susceptibility, insulin resistance due to FFA and tumor necrosis factor -α (TNF-α), an inhibitor of insulin receptor kinase activity, leptin-regulating hormone appetite and adiponectin (43, 44). The inflammatory theory of the diabetogenic process and the chronic complications installed in the context of insulin resistance and obesity is intensely debated, but not fully elucidated (45, 46). Obesity is a major risk factor for T2DM leading to destruction of insulin receptors causing insulin resistance. Obesity is characterized by hypertrophy and hyperplasia of the white adipose tissue and the transformation of “silent” adipocytes into aggressive adipocytes, capable of autonomous inflammation, apoptosis and increased secretion of proinflammatory adipokines (47). The white adipose tissue is producing more than 100 various signal molecules, such as hormones (adiponectin, leptin, resistin, visfatin), chemokines, such as monocyte chemoattractant protein-1 (MCP-1) and proinflammatory cytokines, including TNF-α and interleukin-6 (IL-6) all culminating in a “low grade inflammation” with multiple effects on the endothelial cells (48). There are at least three intracellular signaling pathways linking obesity to inflammation: the proinflammatory kinases pathway, the suppressors of cytokine signaling proteins (SOCS) pathway and the oxidative stress (49). Adiponectin is secreted only by the white adipose tissue (48) and its serum concentration is paradoxically inversely proportional to the body mass index (BMI); numerous studies have clearly revealed its anti-inflammatory and anti-apoptotic roles, as well as its insulin sensitivity (48). In obese individuals the connection between serum leptinemia and the percentage of body adiposity is best observed (47). Leptin has a direct inhibitory effect on both basal as well as glucose-induced insulin level, through different mechanisms, also modulating the expression of secreted and membrane-associated mucins in colonic epithelial cells, targeting protein kinase C (PKC), phosphatidylinositol-3-kinase (PI3K) and mitogen-activated protein kinases (MAPK) pathways (50). Complexity is also evident in the process of transporting glucose into adipocytes; 8 out of the 14 members of the glucose transporters (GLUT) are expressed in adipocytes. The SOCS1, SOCS3, SOCS6 proteins are involved in increasing insulin resistance by inhibiting insulin sensitivity (51).

**Figure 3:**
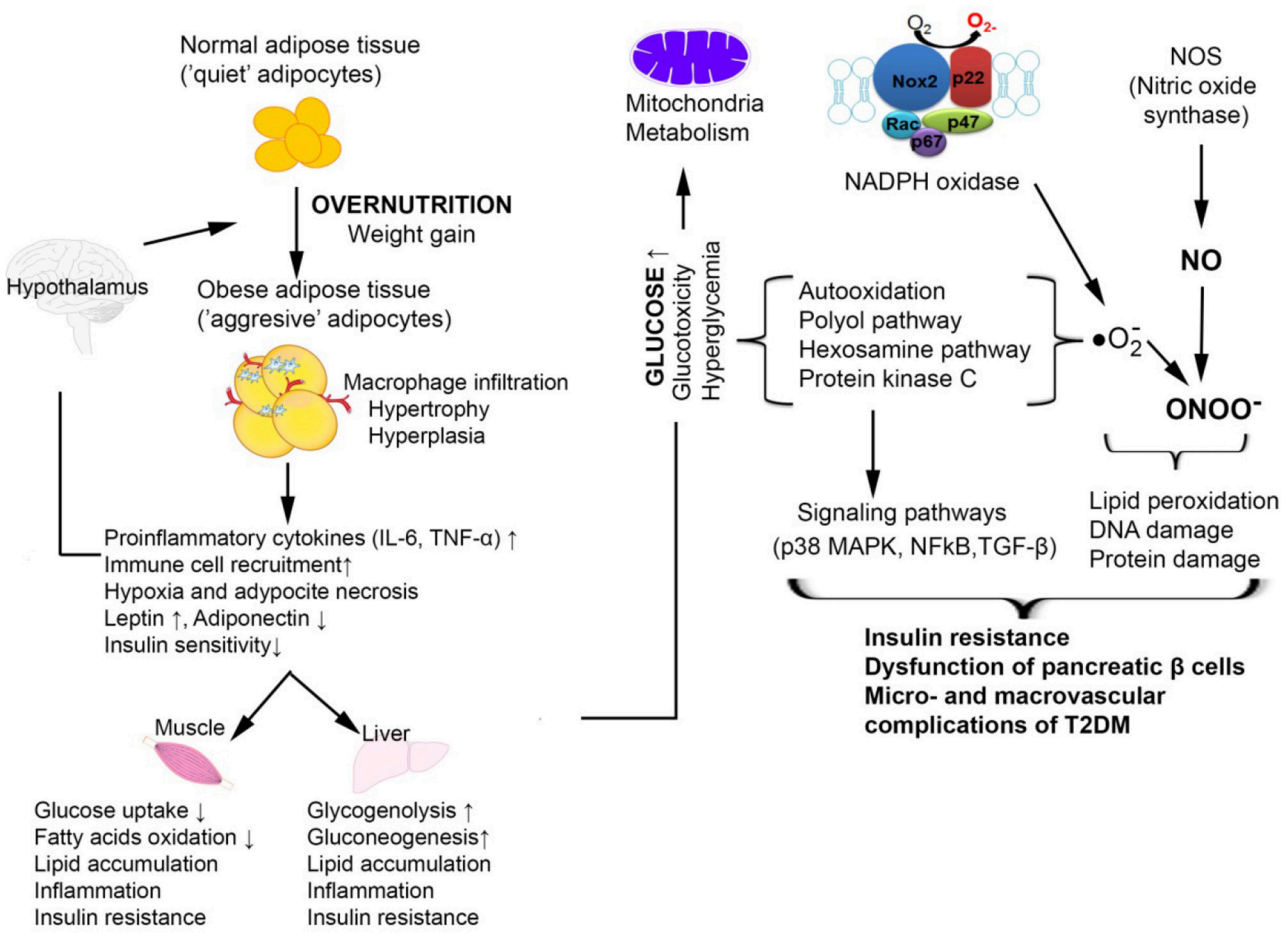
Cellular mechanisms by which oxidative stress is involved in T2DM associated with obesity [adapted after (57)]. In obesity, the expansion of the adipose tissue promotes increased macrophage infiltration and inflammation with increased production of pro-inflammatory cytokines TNFα and IL-6. This is accompanied by an increased release of free fatty acids and an aberrant secretion of adiponectin and leptin. The resulting chronic hyperglycemia determines the activation of glucose self-oxidation processes that lead to the formation of highly reactive hydroxyl radicals which together with nitric oxide (NO) forms peroxinitrite, a very aggressive radical that causes oxidative damage to DNA, proteins, and lipids. The increased oxidative stress also activates signaling pathways such as p38 MAPK, NF-κB which are involved in insulin resistance and vascular complications of T2DM.

The overlapping of metabolic and immune processes can become detrimental under metabolic stress conditions, such as the immunosuppression characteristic of malnourished individuals (52). The adipose tissue releases cytokines that cause up regulation of nicotine-amide-adenine diphosphate oxidase enzyme (NADPH), nitric oxide synthase (NOS), and myeloperoxidase (MPO) from macrophages and adipocytes, favoring the inflammatory processes. Chronic hyperglycemia leads to an activation of glucose self-oxidation processes, formation of highly reactive hydroxyl free radicals and decrease of antioxidant capacity and glutathione regeneration (53). The non-enzymatic glycation processes render proteins more susceptible to oxidative stress processes. Hyperglycemia also stimulates the hexosamine pathway, inducing the formation of fructose-6-phosphate, a protein glycosylation substrate with proteoglycan production, but also changes in gene expression for plasminogen activator inhibitor (PAI) and tumor growth factor -β (TGF-β). Glycation of lipoproteins leads to decreased low density lipoprotein (LDL)-cholesterol catabolism and accelerated catabolism of high density lipoprotein (HDL)-cholesterol, favoring atherosclerosis and micro-/macro-vascular T2DM complications. Increased oxidative stress due to the persistence in circulation of triglyceride-rich lipoproteins along with chronic hyperglycemia is closely related to changes in monocyte and macrophage function (54). Catabolism of glycated proteins leads to the generation of advanced glycation end products (AGEs), stimulating IL-1 synthesis with fibroblast proliferation and endothelial damage. Among the consequences of excess reactive oxygen species (ROS) production is the complex process of lipid peroxidation, the resulting products being extremely unstable. Polyunsaturated fatty acids oxidation cascade is highly activated by free radical molecules (55), leading to the activation of a proinflammatory type processes *via* the mRNA-activated p38MAPK, c-Jun N-terminal kinases (JNK), Janus kinase (JAK), NF-kB, TGF-β, which either directly inhibit insulin activity or lead to a synthesis of inflammatory mediators. The synthesized proinflammatory cytokines stimulate ROS synthesis and intensify oxidative stress (56). The oxidative stress and inflammation, especially when associated with obesity, coexist and mutually potentiate, leading finally to insulin resistance, beta-pancreatic cell dysfunction, and vascular complications of T2DM (Figure 3).

Several studies have associated oxidative stress with dysbiosis. The intestinal tract has a radial oxygen gradient hence microbes residing on the colonic mucosa harbor elevated oxygen tolerance and catalase expression compared to luminal or stool-associated bacteria (58). In addition, inflammation usually promotes an oxidative state which enhance the enrichment of aerotolerant phyla such as Actinobacteria and Proteobacteria. Intestinal inflammation has also been shown to increase the production of terminal electron acceptors for facultative anaerobes such as Enterobacteriaceae further augmenting dysbiosis in the intestine (59, 60).

### Microbial Signatures in T2DM and Obesity

The disruption of normal microbiota (generally referred to as dysbiosis) has been described to be involved in a large spectrum of diseases, including diabetes, obesity, and insulin resistance (61), through disturbing the energy balance (by increasing the metabolic rate; coordination of choline availability, affecting indirectly the liver storage of triglycerides; inhibition of fasting-induced adipose factor—FIAF expression which promotes triglyceride deposition in adipocytes; production of SCFAs which increase lipogenesis, inhibit fatty acids oxidation and trigger the intestinal absorption of monosaccharides by stimulating GLUT1 expression, modulation of YYP and GLP-1 hormones, modulation of intestinal motility and permeability, activation of Toll-like receptors, etc. (62–65).

It has been therefore suggested that the modulation of microbiota, either directly (by antimicrobials, diet, prebiotics and/or probiotics, stool transplant, microbial-derived signaling molecules or metabolites) or indirectly (e.g., immunotherapy) may contribute to the therapeutic management of these pathologies (66).

The impact of the intestinal microbiome on the pathophysiology of diabetes was revealed by several diabetes prone animals, specifically non-obese diabetic (NOD) mice and bio-breeding diabetes prone (BB-DP) rats and human studies. An initial study revealed that NOD mice with a chronic viral infection harbored a lower diabetes incidence (67). Mycobacterial infection followed by stimulation with bacterial antigens decreased the incidence of diabetes development in NOD mice, whereas a germ-free environment increases the risk of diabetes development (15). Nevertheless, recent studies showed that rather certain microbiota members (i.e., *Bacillus cereus*) were responsible for modulating the risk of diabetes development (68). A study by Brugman et al. (69), that used BB-DP rats and fluorescence *in situ* hybridization specific for 16S rRNA of *Clostridium* sp., *Bacteroides* sp. and *Lactobacillus* sp., revealed that rats developing diabetes were colonized by higher levels of *Bacteroides* sp. (69). Further studies highlighted that BB-DP rats harbored a microbiota characterized by lower levels of *Bifidobacterium* sp. and *Lactobacillus* sp. In comparison to control, diabetes free rats. Patterson et al. employed the streptozocin (STZ)-induced T1DM rat model to gain insights into the diabetes onset and progression in terms of microbiota shifts (70). T1DM was associated with changes in the Bacteroidetes: Firmicutes ratio while later T1DM progression was defined by elevated acid bacteria (i.e., *Lactobacillus* sp., *Bifidobacterium* sp.).

T1DM prone rats showed increased gut permeability and altered levels of the tight junction proteins claudin zonulin (71, 72). Within this context, a study employing the BB-DP rat model suggested that administration of *Lactobacillus johnsonii N6.2* hindered diabetes development through regulation of gut integrity, specifically by modulating the tight junction protein claudin-1 (73). The knock out (KO) of myeloid differentiation primary response 88 (Myd88) (an adapter protein downstream of multiple TLR involved in sensing of microorganisms) in the NOD mouse protected against diabetes. Notably, heterozygous MyD88KO/+ NOD mice, which normally develop disease, were shown to be protected from diabetes when they were colonized from birth with the intestinal microbiota of a MyD88-KONOD donor mouse (74). Hence, disease progression in the NOD mouse is partially caused by an aggravated innate immune response to commensals and microbiota changes may counteract disease.

It has been shown that the altered microbiota of genetically obese mice is enough to promote increased adiposity in lean mice that receive a microbiota transplant. Moreover, the gut microbiota of obese people can lead to obese or adiposity phenotypes when transferred to mice (75).

**Figure 4:**
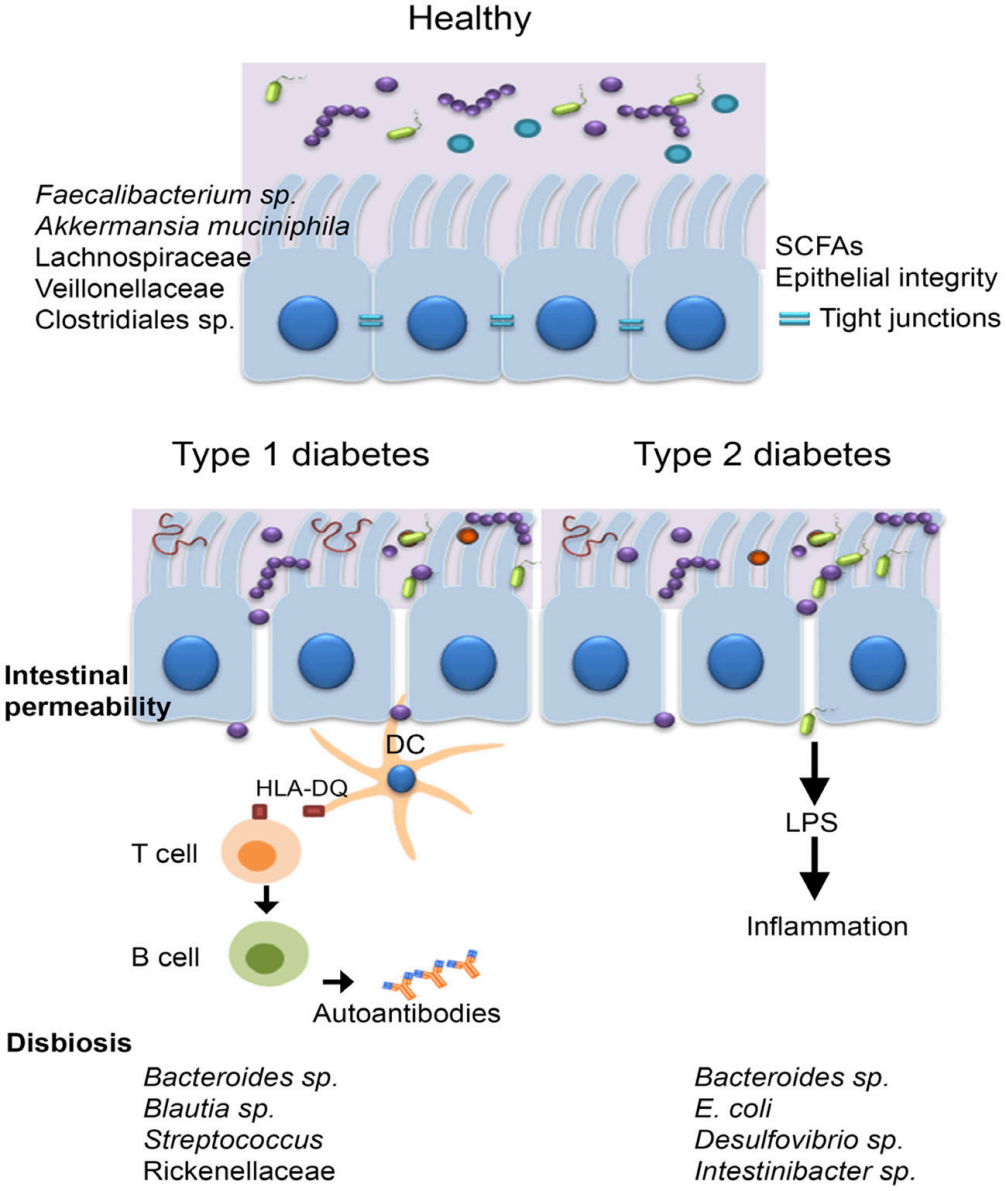
The microbiota of diabetic patients. Individuals with diabetes were reported to have an impaired gut barrier function, characterized by a thinner mucus layer, and increased intestinal permeability. T1DM is an autoimmune disease caused by T-cell–mediated destruction of insulin-producing β-cells, the main genetic predisposition being the human leucocyte antigen (HLA) DR3-DQ2 and DR4-DQ8 haplotypes. Other factors including diet, antibiotic administration, infections, birth delivery mode have all been linked to T1DM development, however the mechanisms involved are not clear. The microbiota of T1DM patients is enriched in bacteria such as *Bacteroides* sp., *Blautia* sp., *Streptococcus* sp., and *Rikenellaceae* and low in butyrate producing bacteria, such as *Faecalibacterium prausnitzii* and mucin degrading bacteria (i.e., *Akkermansia muciniphila*). In case of T2DM, disease is generally triggered by lifestyle choices (unbalanced diet characterized by a high intake of refined sugar and saturated fats, sedentarism); individuals with T2DM harbor a microbiota characterized by elevated levels of *Bacteroides* sp., *Intestinibacter* sp, *Escherichia coli*, and *Desulfovibrio* sp.

Research carried out within the last few years has highlighted the features of the human diabetogenic microbiota (Figure 4).

As suspected, the studies demonstrated that microbiome profiles in diabetics were depending on ethnic origin, diet, geography, and age. However, in spite of these variations, some hallmarks could have been established. All research studies have highlighted *Bacteroides* sp. as a main culprit for type 1 diabetes mellitus (T1DM) associated dysbiosis (76).

It has been proved that *Akkermansia muciniphila* is more abundant in healthy individuals with normal weight than in individuals with obesity and T2DM (25). Furthermore, the Bacteroidetes: Firmicutes ratio was suggested as an early marker for autoimmune diseases since an enrichment of Bacteroidetes was reported in children who developed T1DM (77), but also for obesity and impaired glucose metabolism (78).

Individuals with both T1DM and T2DM were shown to be colonized by low levels of butyrate producers including *Faecalibacterium prausnitzii, Roseburia intestinalis, Clostridium* sp., *Eubacterium rectale*, and *Faecalibacterium* sp. (79) and of mucin degrading bacteria such as *Akkermansia* sp. and *Prevotella* sp. (77, 80, 81). A study that analyzed Scandinavian post-menopausal women revealed lower levels of *Faecalibacterium prausnitzii* and *Roseburia intestinalis* in T2DM compared with individuals having impaired glucose tolerance.

Kostic et al. (82) analyzed 33 infants from Finland and Estonia who were genetically predisposed to diabetes and detected a relative 25% reduction in alpha-diversity in T1DM children but not in disease free seroconverters (82).

T1DM subjects were shown to have a microbiota with an elevated level of “pathobionts” that are commensal bacteria with a pathogenic potential, such as members of *Blautia* sp., *Rikenellaceae*, *Ruminococcus* sp. and *Streptococcus* sp. In addition, a depletion of Lachnospiraceae and Veillonellaceae, commonly encountered in inflammatory conditions was also evident. Recently, De Groot et al. (83) showed that fecal samples of T1DM patients were enriched in *Christensenella* and *Bifidobacterium* and low in SCFA producers like *Roseburia* (83). The human T2DM intestinal microbiota was shown to be inhabited by opportunistic pathogens including the sulfate-reducing genus *Desulfovibrio, Escherichia coli*, and *Bacteroidaceae*.

**Figure 5:**
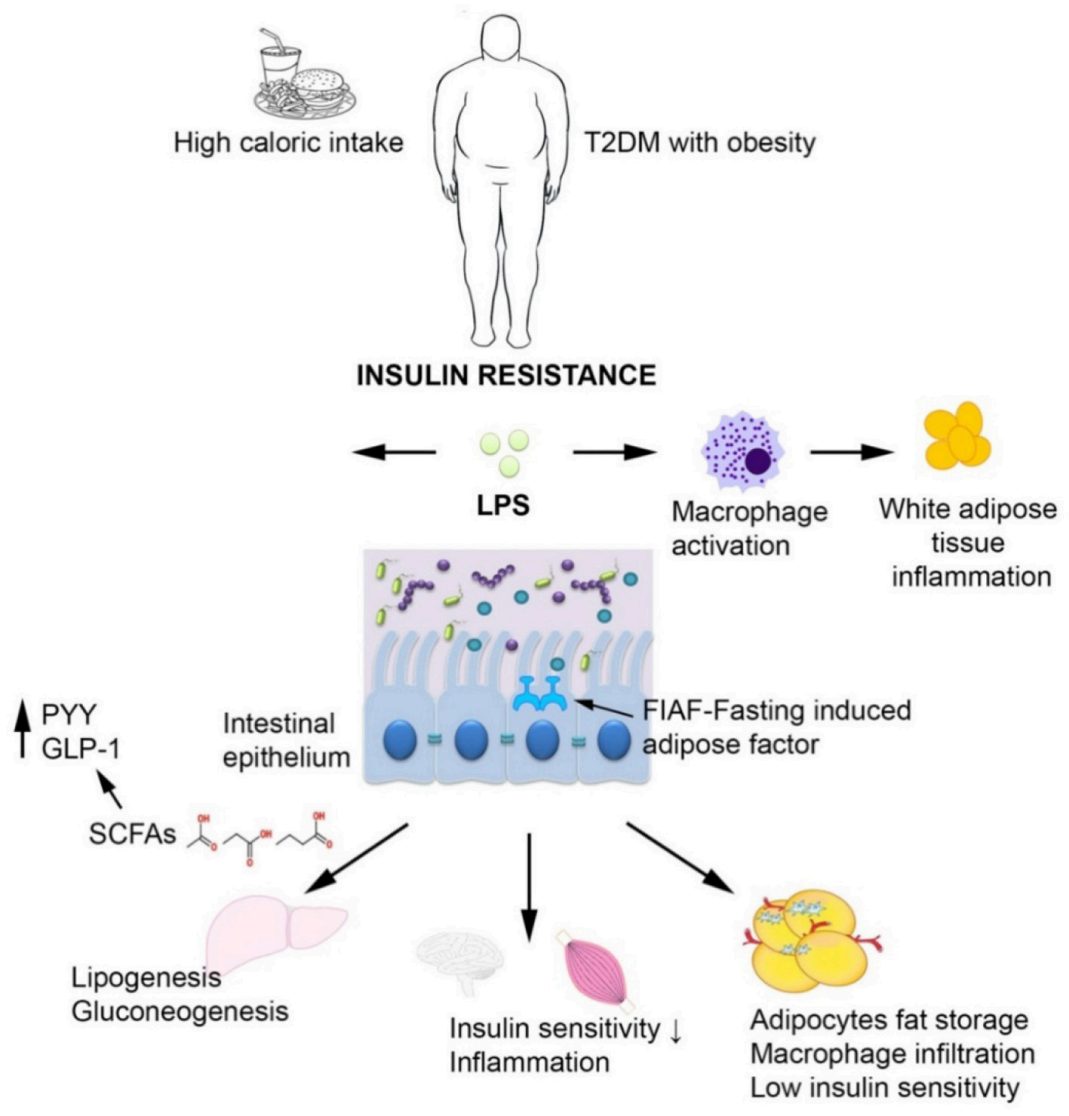
The interplay between T2DM, obesity and the intestinal microbiota. In case of T2DM and obesity, increased intestinal permeability may trigger chronic bacterial translocation which in turn leads to systemic inflammation characterized by macrophage influx into the visceral adipose tissue, insulin resistance, and hepatic Kupffer cells activation. The short-chain fatty acids produced by the microbiota normalize gut permeability and reduce free fatty acid production.

A Chinese study reported an increase of *Escherichia coli* in T2DM patients while a Danish study reported elevated Proteobacteria in T2DM (84). The bacterial counts of the *L. acidophilus, L. plantarum, and L. reuteri* subgroups of *Lactobacillus* sp. were significantly lower among Romanian patients with T2DM and obesity compared to healthy controls (85). A study performed on Romanian subjects revealed that the most common aerobic/facultative anaerobic species isolated from the stool cultures of 100 patients with metabolic syndrome, such as dyslipidemia, diabetes, and obesity were the Gram-negative (86). These Gram-negative bacteria may be linked to T2DM pathophysiology through the release of lipopolysaccharides (LPS) that promote a subclinical proinflammation which typical to diabetes and obesity. The so-called metabolic endotoxemia was firstly described in mice (87, 88) and defined as increased plasma levels of LPS, which are first bound by CD14 (cluster of differentiation 14), then recognized by the Toll like receptor 4 (TLR4) and thereafter, the formed LPS/CD14/TLR4 complex is taken up into chylomicrons, promoting systemic inflammation and subsequently inducing insulin resistance (2, 75, 89). Bacterial peptidoglycans and LPS are also recognized by specific receptors of intestinal dendritic cells, such as Nucleotide-binding oligomerization domain-containing protein 1 (NOD1), CD14 and TLR-4, inducing mucosal inflammation and bacterial translocation, by activating the nuclear factor kB (NF-κB) pathway (90). The term of metabolic infection has recently emerged in order to describe the role of the microbiota endotoxemia associated inflammation together with insulin resistance in T2DM (91). Another mechanism of increased intestinal permeability is represented by the reduced expression of epithelial tight junction proteins, including zonulin occluding (92). Zonulin levels are associated to changes in tight junction and altered intestinal permeability, allowing the invasion of antigens from intestinal environment, leading to inflammation and increased oxidative stress (62) (Figure 5).

Recent molecular studies indicated the presence of *Methanobrevibacter smithii* in the human gut, with variable prevalence, increasing with age, whereas a meta-analysis showed that the obese individuals had a fewer *Methanobrevibacter* sp. than non-obese ones. These results imply that obesity is associated to a different gut microbiota pattern (75).

Furthermore, several studies have characterized gut microbiota in obese subjects. The Roux-en-Y gastric bypass (RYGB) is a powerful treatment for weight loss and improvement of metabolic status. Furet et al. (93) have analyzed the gut microbiota in fecal samples before and after RYGB. The *Bacteroides/Prevotella* group had a low abundance in obese subjects before and increased 3 months (M3) after RYGB; the abundance of *Escherichia coli* cells also increased at M3 and was inversely correlated with leptin levels and fat mass regardless of food intake. Also, lactic acid bacteria including *Lactobacillus/Leuconostoc/Pediococcus* group and *Bifidobacterium* sp. and were lower at M3 (93).

It has been demonstrated that stool transplant from a lean, insulin-sensitive donor to insulin-resistant obese men showed that insulin sensitivity of a subset of participants was significantly improved 6 weeks after the transplant (94).

### Influence of Diet on Gut Microbiota in Diabetes and Obesity

Diet is one of the major lifestyle factors involved in the genesis, prevention and control of diabetes, obesity and other cardiometabolic diseases, being also strongly linked to changes in microbiota. Many reports have shown that the genetic susceptibility to obesity may have interacted with an obesogenic environment (e.g., a major shift in dietary patterns influencing the gut microbiota, a sedentary lifestyle and physical inactivity) in determining the obesity epidemic (23). To date, there are many popular diets including Mediterranean, gluten-free, vegan, Western, omnivore, vegetarian. To date, most of these diets have been clearly linked to different microbiome profiles (95). For instance, gluten free diet is characterized by low *Bifidobacterium* sp. and *Lactobacillus* sp., whereas pathobionts, such as *E. coli* and total *Enterobacteriaceae*, increased proportionally with the reduction in polysaccharide/fibers intake (96). In addition, a short-term gluten-free diet lead to diminished levels of *Ruminococcusbromii* and *Roseburiafaecis* and increased *Victivallaceae* and *Clostridiaceae* (17, 33).

The Western lifestyle countries, following the industrial revolution, underlined a nutritional transition from the traditional diet to a diet rich in heavily processed foods, fats, sugars, proteins, plus different additives, while remaining low in micronutrients and dietary fibers (also referred to as Western diet). Dietary fibers are essential for gut health due to their role in stimulation of the growth and/or activity of certain beneficial microorganisms (97). People in traditional societies, with a fiber intake of almost 50–120 g/day harbor a much more diverse gut microbiota, on its turn indicating a “good health” condition. SCFAs are found in lower amounts in individuals consuming a Western diet. Plant fibers are rich sources of polyphenols that are considered redox mediators, regulating the oxygen tension in the gut lumen, where it is considerably lower, in comparison with that of the gut mucosa, with sufficient high oxygen tension to inhibit growth of strictly anaerobic microbes; in the lumen the anaerobes are favored by high consumption of these non-digestible carbohydrates. A fiber-rich diet leads to an increase of microbial genes in obese patients (98). Several studies reported that a diet enriched in non-digestible carbohydrates modifies the gut microbiota by increasing probiotic bacteria such as bifidobacteria and lactic acid bacteria. Indeed, diets rich in whole grain and wheat bran promoted an increase in intestinal bifidobacteria and lactobacilli (99, 100). In addition, resistant starch and whole grain barley increased the abundance of *Ruminococcus* sp., *E. rectale*, and *Roseburia* sp., whereas FOS, polydextrose- and AOS-based prebiotics were shown to reduce *Clostridium* and *Enterococcus* species (95).

Western diet was correlated with a decrease in the total bacterial load and in beneficial commensals, such as *Lactobacillus, Bifidobacterium* and *Eubacterium* species. Conversely, subjects consuming vegan and vegetarian diets which are rich in fermentable plant-based foods were reported to have a microbiota characterized by a lower abundance of *Bacteroides* sp. and *Bifidobacterium* sp. (101).

The Mediterranean diet (vegetables, moderate consumption of poultry, olive oil, cereals, legumes, winenuts, fish and a low amount of red meat, dairy products, and refined sugars) provides beneficial effects through the elevated content in mono-unsaturated and poly-unsaturated fatty acids, as well as high levels of antioxidants, fibers and vegetable protein content (102, 103). The gut microbiota in individuals receiving Mediterranean diet is characterized by a high colonization by *Lactobacillus* sp., *Bifidobacterium* sp., and *Prevotella* sp., and low levels of *Clostridium* sp. (95), species which are associated with weight loss, improvement of the lipid profile and decreased inflammation.

Dietary proteins were reported to be involved in shaping the microbiota since 1977. Thus, individuals consuming a diet rich in beef had high levels of *Bacteroides* sp. and *Clostridia* and were low in *Bifidobacterium adolescentis* unlike individuals eating a meatless diet (104). Several studies have recently shown that diets including vegetarian whey/pea protein, and animal protein (meats, eggs, and cheese) are linked with microbial diversity (95). Consumption of animal-based protein was positively associated to a richness in bile-tolerant anaerobes, including *Alistipes* sp., *Bilophila* sp., *and Bacteroides* sp. (105).

As shown by animal studies, a high fat diet leads to the establishment of a microbiotalow in *Lactobacillus intestinalis* and high in *Clostridiales, Bacteroidales*, and *Enterobacteriales* (106). Studies in mice revealed the effects of different type of lipids on the microbiota. While lard-fed mice harbored increased numbers of *Bacteroides* sp. and *Bilophila* sp. and reduced levels of *Desulfovibrio* sp., those fed with fish oil had increased lactic acid bacteria (*Lactobacillus* sp. and *Streptococcus* sp.), Verrucomicrobia (*A. muciniphila*), and Actinobacteria (*Bifidobacterium* sp. and *Adlercreutzia* sp.) and mice fed with a diet rich in milk fat or supplemented with taurocholic acid (a biliary acid) showed increased levels of *Bilophilawadsworthia* (107). The fact that the type of fat used in one's diet has distinct impact on the microbiota was recently analyzed in a study by Prieto et al. (108). Indeed, a diet enriched with extra virgin olive oil (EVOO) harbored a different effect on the intestinal microbiota in comparison with an enriched butter diet (BT). Swiss Webster mice fed with BT exhibited the highest values of systolic blood pressure and increased abundance of *Desulfovibrio* whereas mice fed EVOO had the lowest values of plasmatic insulin and leptin and a significant increase in *B. fragilis* (108). It has also been shown in mice that shifting from a high-fat/low-fiber diet to a low-fat/high-fiber diet can alter microbiota composition within a day. Importantly, diet also depends on the enterotype, as subjects on a diet high in animal fat/carbohydrate-rich have *Bacteroides*-/*Prevotella-*dominated enterotypes (109). Mice receiving high fat diet (HFD) until diabetes occurrence, developed endotoxemia, increased gut permeability and microbiota changes (110). The level of plasma endotoxins were higher in mice raised in conventional conditions fed with saturated fatty acids in comparison to animals fed with polyunsaturated fatty acids. Moreover, lard-fed mice exhibited white adipose tissue inflammation and impaired insulin sensitivity compared to fish oil-fed mice (111). In the adipose tissue, endotoxins activate TLRs, inducing the expression of chemokines required for macrophage infiltration (112). It is well established that the intestinal microbiota interacts with the innate immune system to trigger adipose inflammation, since mice deficient in TLRs signaling (through loss of the adaptor proteins MyD88 or TRIF-TIR-domain-containing adapter-inducing interferon-β) showed reduced levels of inflammation in adipose tissue and were protected from saturated fatty acids induced insulin resistance (111, 113, 114). Mice deficient in Myd88, but not for TRIF, are protected from diet-induced obesity, suggesting that obesity and insulin resistance are controlled by different mechanisms. The insulin resistance promoted by high-fat diet is correlated with increased levels of Th17 cells, involving gut microbiota, by the following mechanism: the fat-induced restriction of the commensal segmented filamentous bacteria determines the expression of IL-23 in enterocytes (115); IL-23 activates the release of IL-22 from innate lymphoid cells in the submucous ileal lymphoid folicles; IL-22 induces the production of the serum proteins amyloid A1 and A2, which are required for the activation of Th17 cells in the ileum. Unlike fat, fructose and glucose induce increased levels of diacylglycerols (DAG) which are mediators for insulin resistance in the liver. Ramos-Romero et al. demonstrated that GIT microbiota of rats receiving an excess of fats and sugars in their diet has shown elevated levels of Enterobacteriales and *Escherichia coli*, the combination of fats and sugars proving more harmful than fat or sugar alone when taken in excess (116).

A high protein consumption elevates insulin-like growth factor 1 (IGF-1), proved to be linked to a high diabetes risk (117). Ley et al. (78) found that genetically obese ob/ob animals fed with polysaccharide-rich diet have a 50% decrease in the quantity of Bacteroidetes and a proportional Firmicutes increase in cecal microbiota, this trait being transmissible.

Comparative studies performed on germ-free and conventional mice have demonstrated that after receiving a Western-style diet, germ-free mice exhibited reduced adiposity and improved insulin and glucose tolerance compared to conventional counterparts (118). The colonization of germ-free mice with microbiota from conventionally-raised mice lead to a significant increase in insulin resistance and body fat (64).

### Diet-Microbiota Interactions in Patients With Diabetes and Obesity

The role of diet in diabetes was highlighted decades ago by the observation that this disease was highly prevalent among rich people with easier access to food, such as white flour, refined sugar, and oils, animal fats (±fried) (119).

Many research studies have highlighted a strong link between high sugar intake and T2DM development (120). Based on their capability to be degraded enzymatically in the small intestine, carbohydrates are either digestible (i.e., starch and sugars including glucose, lactose, fructose, sucrose) or non-digestible (resistant starch and fiber). After degradation, digestible carbohydrates release glucose into the bloodstream and lead to an insulin response (95). Their ingestion in high levels leads to a microbiota enriched in *Bifidobacteria* and low in *Bacteroides* (121). The addition of lactose to the previously mentioned diet replicated the same bacterial shifts but also decreased the levels of *Clostridia* (122). The high levels of fructose corn syrup used for the production of soft drinks increased the blood glucose levels and the body mass index thus linking the intake of soft drinks with obesity and T2DM (123). Moreover, diet soft drinks were reported to contain various glycated chemicals which promote increased insulin resistance (124). Ludwig et al. studied 500 ethnically diverse children for a period of 19 months and showed that the obesity frequency increased for every intake of carbonated soft drinks (125).

Artificial Sweeteners are still an intense subject of debate in the carbohydrate fields. Artificial sweeteners such as sucralose, saccharin, and aspartame were designed to be a healthier food additive aimed to replace natural sugar. However, recent work by Suez et al. showed that artificial sweeteners can lead to glucose intolerance faster than the glucose or sucrose. The effects produced by artificial sweeteners were attributed to the increased abundance of *Bacteroides* sp. and decreased *Lactobacillus reuteri* (126).

Contrary to digestible carbohydrates, non-digestible carbohydrates are digested in the colon where they undergo fermentation by commensals leading to the production of SCFAs. It has been shown that *Faecalibacterium prausnitzii*, a high SCFA producer is depleted in diabetic patients (127). When fed with high levels of resistant starch, individuals who did not harbor high *Ruminococcus bromii* also had the highest levels of undigested starch in the stool, suggesting that the microbiota composition determines carbohydrates accessibility to the microbiota. Cocoa products rich in polyphenols (e.g., flavonol) have been shown to help preventing cardiometabolic disorders, exhibiting antioxidant, anti-atherogenic, anti-depressant, anti-inflammatory, antihypertensive, and anti-thrombotic effects, as well as influence on vascular endothelial function, insulin sensitivity and activation of nitric oxide (with protective role by relaxing the blood vessels and lowering blood pressure) (128). Clinical trials reported that dietary polyphenols increase the levels of *Bifidobacterium* sp. (129). Indeed, daily consumption of red wine polyphenols for 4 weeks significantly increased *Bifidobacterium* sp., *Prevotella* sp., *Bacteroides* sp., *Eggerthellalenta*, *Enterococcus* sp., *Bacteroidesuniformis*, and *Blautiacoccoides*-*Eubacteriumrectale* abundance compared with baseline (130). It has been shown that chocolate or cocoa reduced insulin and fasting insulin after glucose challenge and improved insulin resistance, while there was no change on fasting glucose and glycated hemoglobin (HbA1c) (131). Consumption of dark chocolate containing 500 mg polyphenols for a period of 4 weeks lowered fasting glucose, blood pressure, and insulin resistance compared to 20 g of placebo dark chocolate with a negligible polyphenol content (132). Drinking cocoa flavanols (902 mg) for 12 weeks also enhanced insulin sensitivity in overweight and obese people compared to a low-flavanol cocoa drink (133). However, daily consumption of 25 g dark chocolate for 8 weeks did not improve insulin, fasting glucosea and HbA1c levels in hypertensive diabetics compared to those who served 25 g of white chocolate (134). Several studies have reported the positive effects of cinnamon on glycemic control. Importantly, cinnamon contains polyphenols (e.g., cinnamtannin, trans-cinnamic acid procyanidin), flavones (cinnamaldehyde and trans-cinnamaldehyde), and catechin (129). Even though clinical studies showed positive effects of cinnamon on fasting blood glucose levels, there were no significant changes in HbA1c, HDL, LDL or total cholesterol (135, 136). Other reports showed no significant changes in fasting glucose, HbA1c or insulin levels in the case of 43 subjects with T2DM receiving 1 g of cinnamon daily for a period of 3 months (137), 25 post-menopausal women with T2DM taking cinnamon (1.5 g/day) for 6 weeks (138), 11 healthy subjects taking cinnamon (3 g daily) for 4 weeks (139), and in 72 adolescents with T1DM taking 1 g of cinnamon daily (140). A clinical trial using 58 subjects with T2DM reported that 2 g of daily cinnamon intake for a period of 12 weeks significantly reduced HbA1c and blood pressure (141). Supplementation with 500 mg olive leaf extract for 14 weeks inT2DM patients significantly reduced HbA1c and fasting insulin but had no impact on post-prandial insulin levels (142). Resveratrol supplementation in obese men for 1 month reduced glucose, insulin resistance index and leptin level and lowered inflammatory markers (e.g., TNF-α, leukocytes). Even though resveratrol supplementation also reduced plasma fatty acid and adipose tissue lipolysis in the post-prandial state (143), the study lacked some of the necessary controls; therefore more investigations are required in order to prove that resveratrol holds antidiabetic effects.

Whole grains including soy, rye, wheat, and flaxseed and nuts such as almonds, pecans, and hazelnuts are also a high source of polyphenols (144). Whole grain intake was linked to a decreased risk of T2DM but the mechanism of the protection is not well known (145). Moreover, individuals show large variations in glucose metabolism in response to an intervention based on whole grains. Thus, eating whole grains for a period of 3 days improved glucose tolerance in some responder subjects that revealed an increased prevalence of certain glycoside hydrolases within their microbiome compared to the non-responder subjects. This suggests that the gut microbiota may need to already hold the ability to degrade specific complex dietary carbohydrates. Importantly, individuals whose microbiota a responded to a whole-grain intervention had the tendency to have high fiber diets. Thus, the complex carbohydrates linked to whole grains and metabolically accessible to the responders microbiota were probably inaccessible to non-responder individuals who also did not usually consume diets rich in fiber. Improved glucose tolerance in case of the responders may be due to the enrichment of *Prevotella* sp.. *Prevotella* sp. may also improve glucose metabolism in mice fed diets rich in carbohydrates instead of high-fat diets (146).

Olive leafs and extra virgin olive oil are a source of polyphenols, such as hydroxytyrosol and oleuropein with beneficial effects in T2DM (130).

The Mediterranean diet supplemented with nuts or virgin olive oil harbored anti-inflammatory effects by decreasing chemokines, IL-6, T-lymphocytes, monocytes and adhesion molecules (147). A study performed on patients with high cardiovascular risk showed that a Mediterranean diet rich in extra virgin oil led to a 40% decrease of T2DM risk compared to the control (148). Addition of olive leaf polyphenols ameliorated insulin sensitivity and pancreatic β-cells secretory capacity after oral glucose challenge in middle aged overweight men with risk of developing metabolic syndrome (149, 150).

Several studies in humans indicate that following a vegan diet for at least 6 months or a high-fiber–low-fat diet for 10 days were insufficient to substantially increase microbiota diversity or production of fecal SCFAs.

Even though it may promote a greater weight loss, a protein rich diet can also be detrimental. In this sense, individuals on a high protein/low carbohydrate diet harbored a microbiota with diminished levels of *Roseburia* sp. and *Eubacterium rectale*, but low levels of butyrate in their feces (151). In addition, elevated intake of red meat has been linked to elevated levels of the proatherogenic microbial metabolite TMAO (152). Also, its precursors such as betaine was also associated with cardiovascular diseases and T2DM.

Animal-based diets are also high in fat. An association between fat intake and the subsequent risk of developing T2DM has been reported. A study which enrolled more than a thousand individuals without a prior diagnosis of diabetes reported a relationship among T2DM, fat intake, and impaired glucose tolerance. Consumption of high saturated and trans- fat diets elevates cholesterol levels and is linked with a risk of cardiovascular disease, Conversely, mono and polyunsaturated fats lower the risk of chronic disease development (153). Importantly, high-fat diets enrich the abundance of *Bacteroides* sp. as well as of total anaerobic microorganisms (109, 154). Consumption of a low fat diet leads to the over-abundance of *Bifidobacterium* sp. and a reduction of fasting glucose and total cholesterol, while a high saturated fat diet determines a microbiota enriched in *Faecalibacterium prausnitzii* (155).

### Nutrigenomics and Nutrigenetics in the Context of the Gut Microbiota-Host Metabolism–Diet Trialogue

Recently, nutrigenomics and nutrigenetics have emerged due to the need for a holistic pathway combining genetics, nutrition, metabolism, and “omic” technologies to analyze the intricate relationships between environmental factors relevant to metabolic health and disease status. These emergent fields of nutritional science hold promise in advancing nutrition for optimal individual and public health, being carefully supervised by the International Society of Nutrigenetics/Nutrigenomics (156). Nowadays, personalized nutritional interventions are regarded as crucial factors in preventing or reversing the epidemic features of T2DM and obesity, as well as of other chronic diseases (157).

Based on current views, personalized nutrition occurs at three levels: (i) conventional nutrition based on general guidelines for population groups based on age, gender, and social determinants; (ii) individualized nutrition that takes into account the phenotypic information regarding the person's current nutritional status (e.g., anthropometry, physical activity biochemical, and metabolic analysis), and (iii) genotype-directed nutrition based on a rare or a common gene variation (156).

Certain food ingredients, environmental pollutants, and drugs can induce epigenetic changes related to the occurrence of diabetes and obesity, transmissible to subsequent generations (30, 158). Animal and human studies have shown that the nutritional status in one generation can change the epigenetic profiles in subsequent generations, thus having an evident effect on children health. Within this line of thought, nutrients such as niacin, flavonoids, folate, selenium, and choline are only a few examples. In addition, high-fat diets and maternal protein restriction produce a negative effect on the epigenetic regulation of genes by altering their DNA methylation status, dictating the quick adaptation to available food. As obesogenic diets, high- in fat and calorie-dense foods are unfortunately the most available for the general population, hence, they will be over-consumed not only by the current generation, but also by the subsequent generations, “instructed” to seek and consume the “most available” diet (158).

Obesogenic diets lead to remarkable differences between siblings despite their identical genotype. Studies on primates or rodents reported that an obesity-promoting, calorie-dense maternal diet epigenetically changed the fetal chromatin structure by producing covalent modifications of histones. Maternal food restriction during pregnancy, as well as the nutritional status of grandparents altered the DNA methylation status of genes involved in the pathogenesis of obesity, diabetes or cardiovascular events in subsequent generations (30, 159). Other studies indicated that weight status (as an indicator of food intake) modified the risk of disease in subsequent generations (160). Maternal obesity- or malnutrition-related exposures at the very early development, even prior conception, usually reveal a positive association with congenital anomalies, related to abnormal methylation patterns (161). Obesity of the father was also linked with aberrant low methylation patterns in the offspring (162).

LMW metabolites and signal molecules generated by the indigenous gut and vaginal microbiota of pregnant women can cross the placenta and reach the fetus, affecting its development, metabolism, cognitive function and body composition in the natal and post-natal periods of life through the epigenetic modulation of gene expression (30). This information suggests that significant modifications in the maternal indigenous microbiota may produce long-term metabolic consequences in the offspring which subsequently may affect adult health and life span as a result of epigenetic modifications. Providing pregnant women with an appropriate diet, prebiotics, probiotics, and/or bioactive supplements (enzymes, relevant cofactors, or their precursors) will restore the intracellular concentrations of signal molecules necessary for the epigenetic modulation of chromatin, DNA, and histones or alteration of post-translated products (163).

Even though dietary interventions impact the metabolic host response in an individual manner, analysis of the microbiome can aid to predict the response and moreover, orient the selection of therapeutic interventions (e.g., probiotic strains able to metabolize specific dietary components toward getting a desired effect) (164).

## Therapeutic Interventions Triggering the Gut Microbiota

While governments and health organizations are engaged to discover treatment options for largely preventable diseases, an emerging research area targeting the microorganisms inhabiting the digestive tractis providing new insights and possible ways for intervention.

### Probiotics

Nowadays, probiotics are highly investigated for their effects on host health and, in addition, intensive research is being done to select novel strains with probiotic potential (165–169). Probiotics have anti-inflammatory, hypoglycemic, insulinotropic, antioxidative, and satietogenic properties, thus they can be employed in the treatment of T2DM and obesity (170).

The multiple protective mechanisms of probiotics in T2DM treatment were reported by several studies using animal models. Oral inoculation (0.05%) or diet supplementation (0.1%) of heat-killed *Lactobacillus casei* in several mouse models including KK-Ay mice, NOD mice, and Alloxan-induced diabetic mice led to reduced plasma glucose level and diabetes development (171, 172). Neonatal STZ-induced diabetic (n-STZ) rats that were fed with a diet containing *Lactobacillus rhamnosus GG* for 9 weeks exhibited lower blood hemoglobin levels and an improved glucose tolerance compared to the control group fed with a conventional diet. The group that received *L. rhamnosus GG* harbored a serum insulin level significantly higher than in the control group just 30 min after glucose loading (173). Feeding NOD mice with the multispecies probiotic VSL#3 led to increased IL-10 secretion in Peyer's patches, pancreas, and spleen and lowered β-cell destruction and inflammation. Several reports highlighted that oral administration of probiotics to diabetic rats significantly improved hyperglycemia, oxidative stress, and dyslipidemia.

In fructose-induced diabetic rats of the use of a probiotic containing *Lactobacillus acidophilus NCDC14* and *Lactobacillus casei NCDC19* significantly decreased glycosylated hemoglobin, free fatty acids, blood glucose, and triglycerides (174). Feeding the same probiotic to STZ-induced rats abolished the oxidative damage induced by STZ in pancreatic tissues through inhibition of nitric oxide release and lipid peroxidation and also enhanced the antioxidant potential of catalase, glutathione, superoxide dismutase, and glutathione peroxidase (175). In alloxan induced diabetic rats, probiotic pre-treatment employing a mixture containing *Bifidobacterium lactis, Lactobacillus acidophilus*, and *Lactobacillus rhamnosus* lead to lower blood glucose and elevated the bioavailability of gliclazide, a sulphonylurea used for treating non-insulin dependent T2DM (176, 177). The antidiabetic effects against insulin resistance of different probiotics may also be due to increased hepatic natural killer T (NKT) cells. Depletion of NKT cells determined enhanced secretion of proinflammatory cytokines, whereas HFD was reported to induce depletion of hepatic NKT cells, thus leading to insulin resistance in C57BL/6 mice. Nevertheless, administration of VSL#3 led to reduced insulin resistance, weight loss, and improved inflammation, by modulating NF-kB (178). In HFD fed C57BL/6 J mice, as well as in STZ-induced diabetic rats, *Lactobacillus plantarum* DSM 15313 and *Lactobacillus reuteri* GMNL-263 lowered glycosylated hemoglobin and blood glucose (179, 180). Dendritic cells (DCs) from NOD mice were stimulated with *Lactobacillus casei, Lactobacillus reuteri*, or *Lactobacillus plantarum* for a 1 day and out of the strains tested, *Lactobacillus casei* induced the highest level of IL-10. Subsequently, when the *Lactobacillus casei*-stimulated DCs were transferred to NOD mice, the recipients revealed a significant delay in diabetes incidence (181).

In HFD fed mice, the administration of *Bifidobacterium animalis* subsp. *lactis 420* improved insulin sensitivity and fasting hyperinsulinemia and also reduced the bacterial translocation to mesenteric adipose tissue, lowering the expression of IL-6, TNF-α, and IL-1β in mesenteric adipose tissue, liver, and muscles (90). A novel study by Lee et al. analyzed the anti-diabetic effects of *Lactobacillus plantarum* Ln4 (Ln4), which was obtained from napa cabbage kimchi. The oral administration of Ln4 reduced epididymal fat mass and weight gain, lowered plasma triglyceride levels and the insulin resistance index in HFD fed mice (182). It has been shown that among probiotic bacteria, only *Lactobacillus plantarum* and several selected strains within the genus *Bifidobacterium* (mostly *Bifidobacterium adolescentis* and *Bifidobacterium pseudocatenulatum*) are capable of *in vivo* folate production. Therefore, the use of folate-producing probiotic strains could more effectively confer protection against inflammation (183). Since there are only a few studies available, the role of probiotics in diabetic human subjects is still largely unknown. Several randomized, double blind, placebo controlled (RDBPC) clinical trials investigated the effects of probiotic administration on antioxidant status, blood glucose, and lipid profile in T2DM. The probiotic intervention group consumed 300 g/day of probiotic yogurt containing 10^6^ CFU/ml *Lactobacillus acidophilus La5* and 10^6^ CFU/ml *B. lactis Bb12* whereas the control group consumed the same dose of conventional yogurt for 6 weeks. The probiotic treatment cohort showed as an increase in the activities of the glutathione peroxidase and a significant decrease in fasting blood glucose. The total cholesterol: HDL ratio and LDL-C:HDL-C ratios were also lower in the probiotic treatment group, compared to the control (184, 185). A randomized clinical trial that enrolled 450 patients with T2DM and hyperlipidemia was recently performed by Tong et al. The group tested the hypothesis that gut microbiota alteration may be involved in the mitigation of T2DM with hyperlipidemia by using metformin and a specifically designed herbal formula. Metformin and the herbal formula were shown to ameliorate T2DM with hyperlipidemia by enriching beneficial bacteria, such as *Blautia* and *Faecalibacterium* spp (186). The effects of maternal dietary counseling during pregnancy were investigated in a RDBPC study by Luoto et al. (187). Within this study, 256 pregnant women were randomized into three treatment groups: a control cohort (control/placebo), dietary intervention with probiotics (diet/ *Lactobacillus rhamnosus GG* and *B. lactis*), and with placebo (diet/placebo). The study revealed that the probiotic supplement increased the colostrum adiponectin concentration compared to the control. A recent study by Kijmanawat et al. revealed that 4 weeks supplementation using a probiotic containing *Bifidobacterium* and *Lactobacillus* in women with diet-controlled gestational diabetes in the late second- and early third-trimester lowered fasting glucose and increased insulin sensitivity (188). A recent clinical study by (189), showed that probiotic supplementation (containing *L. casei* 2 × 10^9^, *Bifidobacterium bifidum* 2 × 10^9^*, L. acidophilus* 2 × 10^9^ CFU/day) for a period of 12 weeks had beneficial effects on biomarkers of inflammation in diabetic patients with coronary heart disease (CHD) as well as on glycemic control, HDL-cholesterol, total-/HDL-cholesterol ratio, and oxidative stress (189). Administration of a multi-strain probiotic supplementation over 6 months as a monotherapy lead to significantly lower HOMA-IR in T2DM patients, with inflammation and an improved cardiometabolic profile (190).

Importantly, patients with diabetes are often faced with major depression leading to more diabetic complications, poorer self-care, and lower medication adherence (191). Within this line of thought, probiotics have been administered to diabetics with depression. Vitamin D and probiotic (Lactocare Zisttakhmir Co) co-supplementation for a period of 12 weeks among diabetic people with CHD harbored positive effects on mental health parameters, HDL-cholesterol level, glycemic control, and total antioxidant capacity (192).

However, other studies state that probiotic use does not provide a significant benefit for the diabetic patients. For example, a randomized, double-blinded clinical trial using *Lactobacillus acidophilus NCFM* in 45 men for a period of 4 weeks reported that there were no changes in the expression of baseline inflammatory markers and in the systemic inflammatory response after probiotic treatment (193). Recently, Kobyliak et al. (194), showed that probiotic therapy consisting of a concentrated biomass of 14 probiotic bacteria genera *Bifidobacterium, Lactobacillus, Lactococcus, Propionibacterium* had a modest effect on insulin resistance in patients with type 2 diabetes (194).

### Prebiotics

As a source of SCFAs, prebiotics may improve glucose tolerance. Prebiotics were also suggested to reduce hypercholesterolemia through lowering of cholesterol absorption as well as by inducing SCFAs production by commensals (195, 196). The intake of the prebiotic inulin (20 g/day) significantly lowered serum triglycerides, increased serum HDL-cholesterol, and decreased serum LDL-cholesterol compared to the control group (197). In addition, normolipidemic individuals consuming daily 18% of inulin without any other dietary restrictions exhibited a lowering in triacylglycerols and total plasma cholesterol as well as elevated fecal concentrations of *Lactobacillus*-lactate (198). In rats, the addition of inulin to diet, increased the fecal excretion cholesterol and lipids compared to the control group, due to a lower cholesterol absorption (199). Other prebiotics including oligodextrans, lactose, resistant starches, lactoferrin-derived peptides, and N-acetyl-chito-oligosaccharides were also reported to have hypocholesterolaemic effects in T2DM patients at risk to develop cardiovascular complications (200). A diet rich in arabinoxylan and resistant starch lead to increased *Bifidobacterium* and butyrate levels in patients with metabolic syndrome (201).

Due to their cost, availability and lack of acute toxicity, dietary polysaccharides may be employed alone or in combinations, to improve human health in various health conditions. Using prebiotics in synbiotic products, besides their role in stimulating the expansion of probiotics, they also inhibit pathogen growth, stimulate immunity, and vitamins synthesis. A RDBPC study that enrolled 20 diabetic volunteers aged 50–60 years, for a time frame of 30 days analyzed the effects of a synbiotic drink (a combination of probiotics and prebiotics) on blood glucose and cholesterol levels. The symbiotic treatment group consisted of patients that consumed 10^8^ CFU/mL *Bifidobacterium bifidum*, 10^8^ CFU/ml of *Lactobacillus acidophilus*, and 2 g oligofructose harbored increased HDL cholesterol, and decreased fasting glycemia; but, importantly, no significant changes were observed in the placebo group (202).

## Conclusions and Perspectives

The complex molecular and cellular interactions established between the gut microbiota and the host, starting immediately or even before birth (as stated in some recent studies), have a key role in host homeostasis. Any disorder of the “healthy” gut microbiome induced by infections, changes in the lifestyle, diet or use of antimicrobial substances can result in a plethora of pathologies, such as chronic gastrointestinal diseases, neurological disorders, autoimmune diseases, allergies, diabetes, obesity, cardiovascular diseases, chronic inflammation or cancer. The high biodiversity within the microbiota may act as an important marker of eubiosis. The capacity to access and remodel the composition and function of the microbiota makes it an attractive target for establishing a link between certain patterns of gut microbiota and physiology/pathology status. This paves the opportunity for development of potential biomarkers, and new tools for prevention, screening and treatment in personalized healthcare strategies. Nevertheless, due to the complexity and individuality of human microbiota, a challenge at present time is the identification of microbial patterns and microbial metabolic pathways specifically linked to health or disease. In this regards it is critical to establish the profile, if any, of a normal gut microbiome.

There are many studies linking an altered gut microbiota with metabolic diseases from obesity to T2DM and cardiovascular diseases. Members of the indigenous microbiota may regulate body weight via the modulation of the host metabolism, immunity and neuroendocrine functions. The gut microbiota provides metabolic functions and is involved in regulating host gene expression, influencing the ability to extract and store energy from dietary sources. Moreover, obese individuals with lower bacterial richness/dysbiosis have been shown to have greater weight gain, while microbiota transplantation of either obese or lean mice in germ-free recipients influences body weight, suggesting that the intestinal ecosystem is a powerful tool for weight management.

However, there are still questions and hypotheses waiting to be confirmed or disapproved by future studies, such as: can intestinal microbiota be a target in the treatment of diabetes and obesity? Can the transplant of human normal, “healthy” intestinal microbiota to another human with dysbiosis or “decompensated” intestinal microbiota be possible as a therapeutic measure?

Prospective studies on large cohorts of participants using high-resolution monitoring of host and microbial parameters assessed by omics approaches are needed to determine if the microbiota is altered prior or after disease onset. A multi-parameter individual approach, including microbiota analysis will provide information regarding the characteristics of people responding positively to a given intervention. A deeper understanding of the gut microbiota could essentially contribute to the management of metabolic health and weight loss paving the way for microbiota-focused precision nutrition.

Experiments of microbiota transplantation from humans to mice could highlight the microbial species responsible for the occurrence of obese phenotype vs. protective species, such as *Christensenellaminuta*, specific metabolic pathways (e.g., biosynthesis of branched-chain amino acids, which linked to impaired sensitivity to insulin vs. SCFAs pathway), microbial metabolites (detrimental vs. beneficial), or diets (high fat vs. high in fruits and vegetables; low vs. rich in fibers).

A better knowledge of the interactions between the host and gut microbiota is also needed in order to increase the predictability of the experimental models. Studies performed exclusively on fecal samples could not be indicative about the processes occurring in the small intestine, which are essential for vitamin absorption. Intestinal microbiota can be directly modulated by the use of probiotics, prebiotics, and even antibiotics. The use of antibiotics in early life is correlated with obesity in both humans and mice. Antibiotics can be used for treating certain GIT ailments, but the microbiota alterations they induce may promote metabolic disturbances, changes in intestinal permeability, susceptibility to infections, especially fungal ones and an increased risk of *Clostridium difficile* infection.

Increased polysaccharides levels are likely to be beneficial for individuals eating a typical Western-style, fiber-poor diet. Beneficial metabolites such as butyrate or the bacteria that produce them could be supplemented pharmacologically, while receptor antagonists or enzymatic inhibitors could be developed for detrimental metabolites, such as TMAO. However, controlled dietary interventions that document the utility of various nutrients, supplements, probiotics, and foods in regulating aspects of the microbiota and human health are required. Dietary interventions need to be individually adjusted to prevent or treat chronic diseases based on the genetic background, food and beverage consumption, nutrient intake, microbiome, metabolome, and other omics profiles.

As a general purpose, we can achieve eubiosis and host health by reducing the antimicrobials consumption and assuring the necessary nutrients, respecting everyone's preferences, by a diverse and balanced diet containing macro- and micro-nutrients.

## Author Contributions

VL designed and wrote the manuscript. L-MD wrote the manuscript. GP designed all the figures, wrote part of the manuscript and submitted the paper. AP, LP, and NC wrote the manuscript. MC drafted and revised the manuscript.

### Conflict of Interest Statement

The authors declare that the research was conducted in the absence of any commercial or financial relationships that could be construed as a potential conflict of interest.
